# Microstructure and Fracture Behavior of Special Multilayered Steel

**DOI:** 10.3390/ma13030789

**Published:** 2020-02-09

**Authors:** Xin Zhou, XiaoKang Zhao, Rui Cao, RuiHua Zhang, Yun Ding, XiaoBo Zhang

**Affiliations:** 1 State Key Laboratory of Advanced Processing and Recycling of Non-ferrous Metal, Department of Materials Science and Engineering, Lanzhou University of Technology, Lanzhou 730050, China; Zx19971230@163.com (X.Z.); dingyunms@163.com (Y.D.);; 2 China Iron and Steel Research Institute Group, Beijing 100081, China; zrhyy@hotmail.com

**Keywords:** multilayered steel, tensile strength, bending strength, Cr_23_C_6_ phases, fracture, crack

## Abstract

In this research, multilayered steel (MLS), which is composed of middle-carbon martensite steel, high-carbon martensite steel, and a pure Ni thin layer was obtained by the accumulative roll-bonding method. The microstructure and mechanical properties of the MLS were investigated by scanning electron microscopy (SEM), Vickers microhardness, tensile, and bending tests. In-situ SEM tensile tests were used to observe the crack initiation and propagation processes during the tensile loading. The results show that the ultimate tensile strength and bending strength of the MLS can reach 946 MPa and 3153 MPa, and the maximum elongation can reach 18%, which is related to the better combined quality of the interface. The middle and larger martensite layer (ML) becomes the weakest link of tensile fracture and interfacial delamination of the MLS during the tensile processes, because there are lots of large hard blocks Cr_23_C_6_ phases distributed in the middle thicker ML layer. Besides, the MLS can withstand larger bending deformation. When the MLS was bent to 180 degrees, neither macro-cracks in the outer side of the bending parts nor interfacial delamination can be found.

## 1. Introduction

Multilayered metal composites consisting of alternating ductile and brittle materials show a unique combination of high fracture toughness and high tensile strength, so they have been widely used in structural applications. Some studies have massively concentrated on the mechanical properties of multilayered metal composites, such as deformation, bending toughness, and fracture toughness [1,2,3,4,5].

Multilayered metal composites can be performed by various manufacturing methods, such as accumulative rolling bonding (ARB), explosive welding and rolling, diffusion joining, casting rolling, etc. Martensitic steel has a higher tensile strength and lower tensile elongation; however, austenitic steel has a higher tensile elongation and lower tensile strength. Several studies have concentrated on obtaining a multilayered steel by combining martensitic steel with higher strength and austenitic steel with higher tensile elongation [4,5,6,7,8,9,10,11,12,13]. In Ref. [6], the multilayered metal composite plate can be manufactured by alternating martensitic steel SS420 and austenitic steel SS304 and using ARB, the results indicated that the composite plate can reach a high tensile strength exceeding 1.2 GPa and good plasticity with 15% uniform elongation. In Ref. [7], the elongation of SUS420/SUS301 multilayered metal composites can reach 25%, and it is larger than that of the monolithic SUS420 with less than 4%. Moreover, the tensile elongation of as-quenched martensite in a multilayered structure consisting of brittle martensitic and soft austenitic stainless steel can reach 50%. Moreover, the dislocation structure at true strains of 0%, 5%, 22%, and 40% are compared with brittle martensite [6]. Recent studies found that the tensile elongation of the multilayered steel composites is concerned with the layer thickness and the fraction of the brittle material, the tensile strength ratio between the constituent materials, the hardening exponent, and the interfacial strength between the brittle and ductile layers [6,7,8]. In Ref. [8], the authors indicated that the fracture mode of the brittle layer has been changed from brittle cleavage fracture to ductile shear fracture with the decrease of the layer thickness from 176 to 40 μm. In Ref. [9], the authors found that the tensile ductility was remarkably improved when the bonding strength increased, and that three types of tensile fracture behavior were directly related to the bonding strength of the interface. Chanseo jeong et al. [10] recently demonstrated the fracture behavior and stress–strain distribution of martensite/austenite multilayered composites. Stress partitioning behavior of multilayered steels during tensile deformation were measured by in-situ neutron diffraction, the results indicated that the deformation mode was classified into three stages: a fully elastic stage where the martensite phase and austenite phase deform elastically, a partially plastic stage where austenite yields while martensite continues to deform elastically, and a fully plastic stage where yielding occurs in martensite phase and austenite phase [11]. In Ref. [12], the bending formability of multilayered composites is evaluated by tensile tests, V-bending tests, and hemming tests. Multilayered metals were able to bear severe bending with no cracks and delamination in the outer layers [12]. In 2017, B.X. Liu et al. [13] fabricated multilayered Q235/SUS304 steel by vacuum hot rolling at different rolling temperatures. The results indicated the increase of the interfacial bonding strength with the increase of the rolling temperature led to the increase of the tensile ductility. 

**Figure 1:**
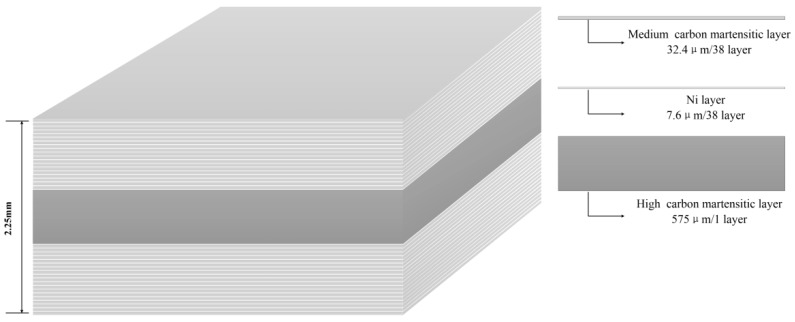
Schematic of as-received multilayered steel.

In the previous works, more studies have concentrated on the manufacturing and properties of three-layer composite plates or multilayer composite plates only by two materials. Based on the difference of the performance requirements, single martensite or austenite stainless steels are usually chosen to obtain three-layer composite plates or multilayer composite plates by combining martensitic steel with higher strength and austenitic steel with higher tensile elongation. However, it is very rare to use more materials to fabricate and study multilayered composite plates. Therefore, the microstructure and fracture behavior of a special multilayered steel with three materials must be investigated. In this study, three different materials are selected for the development of composite plate used in cutting tools. For a middle layer with higher hardness and wear resistance, high carbon martensitic steel is chosen. For the two sides with higher plastic elongation and good strength, the previous works have only used single martensitic stainless steel or a single austenitic stainless steel, depending on the performance requirements. Here, the composite layers including a medium carbon martensitic layer and pure Ni layer are chosen as the two sides, i.e., the composite plate is composed of a middle high carbon martensitic layer and two composite layers at two sides, each composite layer including 19 medium carbon martensitic layers and 19 pure Ni layers, as shown in Figure 1. Therefore, it is crucial to investigate the microstructure and fracture behavior of this composite plate. The microstructures and mechanical properties were characterized, analyzed, and compared by scanning electron microscopy (SEM), electron backscattered diffraction (EBSD), Vickers microhardness, tensile, and bending tests. Through an in-situ SEM tensile test, the propagation of the crack was observed, and the weakest link of the multilayered steel in the tensile process was determined.

## 2. Materials and Methods

**Figure 2:**
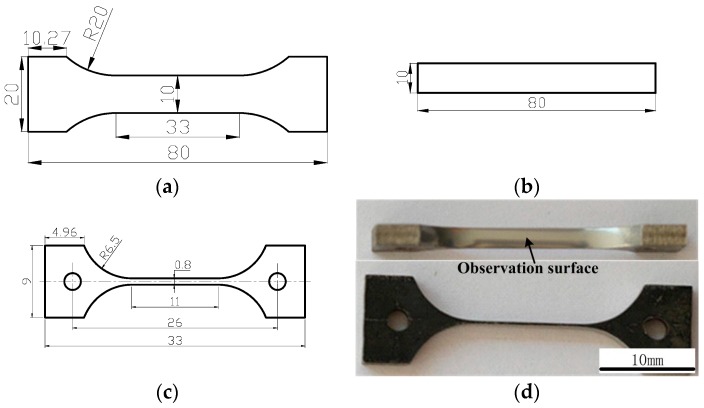
The size of the specimens involved in the experiment (dimensions in mm). (**a**) Dimensions of tensile specimens, (**b**) Dimensions of bending specimens, (**c**) Dimensions of in-situ tensile specimens, (**d**) macro figure of in-situ tensile specimens.

In this study, the multilayered steel that is composed of middle-carbon martensite steel, high-carbon martensite steel, and a pure Ni thin layer was obtained by the accumulative roll-bonding method. In the Introduction, the multilayered steel has been introduced. The thickness of the total multilayered steel (MLS) is 2.25 mm. The chemical composition of each layer in the MLS is shown in Table 1. To reveal the mechanical properties, the tensile tests, bending tests, and hardness measurements were performed. The dimensions are shown in Figure 2.

Tensile experiments were conducted for tensile specimens as shown in Figure 2a at room temperature and the engineering strain rate of 10 × 10^−3^ s^−1^. After the tensile experiments, the strength, ductility, and fracture modes of the composite plates can be obtained. 

Bending experiments were performed using the bending specimens in Figure 2b. The length direction of the specimens was parallel to the rolling direction. The bending span is 28 mm. Three-point bending tests were conducted to the bending angles of 136° and 180° to obtain the bending strength and cracking behavior, respectively. In order to further observe the deformation and the cracking processes of the specimens during the bending process, the side of the bending specimen was first polished and etched so that the microstructure after bending could also be observed by scanning electron microscopy (SEM).

The Vickers microhardness was measured using an automatic hardness tester. Since the thickness of each layer was only 32.4 μm, a small load of 25 g and the dwell time of 15 s were used to ensure the size of indents smaller than 15 μm. The measured result is obtained by the average value of nine indents in the center of each layer.

Especially, for in situ tensile specimens as shown in Figure 2c,d, in-situ SEM tensile experiments were performed in an SEM instrument with an in-situ tensile equipment to characterize the detailed fracture processes. A relatively slow loading rate of 0.033 mm/min (a strain rate of 5 × 10^−5^s^−1^) was used to obtain the deformation, crack propagation, and fracture processes. After fracture, the SEM-observed surface and fracture surface were directly compared. The fracture features were also observed and analyzed by SEM.

To further determine the fracture mechanisms and demonstrate the relationships between the properties and microstructures, the microstructures were grinded, polished, and etched by the etchant reagent with 4 g of picric acid + 5 mL hydrochloric acid + 100 mL ethyl alcohol for 30 seconds, and then, they were observed and analyzed by SEM.

To further clearly characterize the phase and misorientation, an EBSD technique was used in a SEM operated at 20 kV. A 160 μm × 160 μm EBSD area scan was performed using a hexagonal grid with a step size of 0.28 um. 

## 3. Results

### 3.1. Characterization of Microstructure by SEM and EBSD

**Figure 3:**
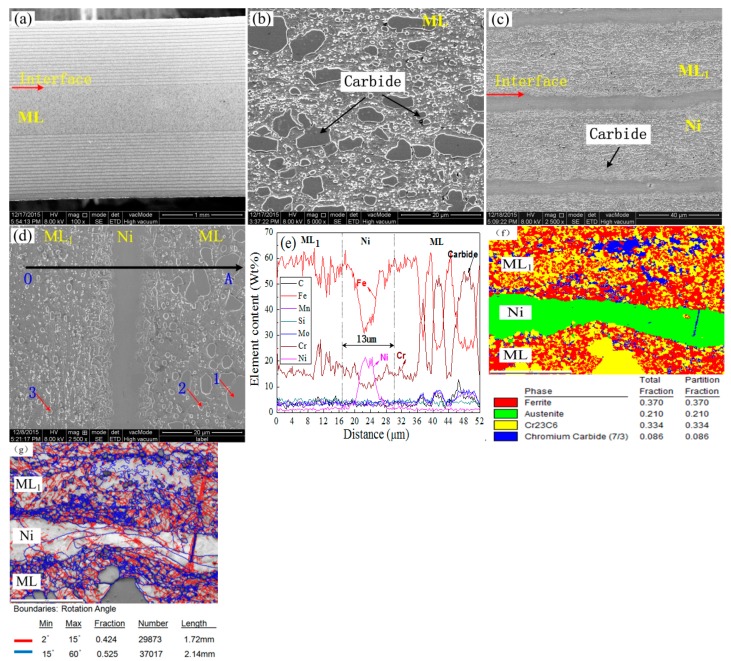
Microstructure and line scanning results of multilayered steel. (**a**) macro-feature of the MLS, (**b**)the magnification of ML in Figure 3a, (**c**) the magnification of the composite layer at two sides in Figure 3a, (**d**) line scanning distribution location, (**e**) line scanning result along the black line OA in Figure 3d, (**f**) EBSD phase analysis, (**g**) the misorientation distribution obtained by EBSD.

Figure 3 presents the SEM microstructure and line scanning distribution of the multilayer specimen. Note that in the following text, as well as in this and later figures, ML denotes the middle martensitic layer and ML_1_ denotes the multiple martensitic layer in the two sides. Figure 3a presents the macro-feature of the MLS used in this paper; the red arrow indicates the interface between the ML and multilayer composites, including ML_1_ and the pure Ni thin layer. The middle and larger martensite layer (ML) in Figure 3a was magnified in Figure 3b. From Figure 3b, the ML is composed of ferrite base metal, Cr_23_C_6_, and Cr_7_C_3_ carbides distributed in the matrix. The composite multilayers distributed in two sides in Figure 3a is magnified in Figure 3c. In Figure 3c, the multilayer composite is alternately composed of 19 middle carbon martensite layers (ML_1_) and 19 pure Ni thin layers. The thickness of each ML_1_ layer and each Ni layer is 32.4 μm and 7.6 μm, respectively. So, the MLS in this paper is composed of a middle ML layer with a thickness of 575 um and two composite layers including 19 ML_1_ and 19 Ni layers. On the two sides of MLS, the thicker ML_1_ and thinner Ni layer were used. To reveal the distribution of the elements between the middle ML layer and the composite layer including the ML_1_ and Ni layers, line scanning distribution along the black line OA in Figure 3d was done in Figure 3e. There is some diffusion of elements Ni, Fe, and Cr in the interface between the ML and Ni layer, and the interface between the Ni layer and ML_1_. Although the C content obtained by EDS is not true, the trend of the change can be seen in Figure 3e. Especially, higher carbon content and higher chromium content are observed in coarse block carbide. From Figure 3f, ML_1_ in Figure 3d is composed of ferrite base metal, Cr_23_C_6_, and Cr_7_C_3_ carbides distributed in the matrix. Cr_23_C_6_ and Cr_7_C_3_ carbides in ML and ML_1_ can be determined by EDS point analysis in Table 2 and EBSD phase analysis in Figure 3e,f. From Figure 3f, it can be also seen that the thickness of Ni layer become non-uniform, which means that different diffusion and deformation can be produced, and in the interface near the Ni layer, some carbides can be found. It is also the result of element diffusion. Figure 3g presents the misorientation distribution obtained by EBSD. Large angle boundaries are distributed at the interface of the Ni layer and ML (ML_1_). In the interfaces, the exist of large angle boundaries will contribute to the slip of the boundaries and the matrix near carbides, which will decrease the stress concentration near carbides, further improving the compatible deformation of grains and boundaries near carbides. The fraction of large-angle boundaries (larger than 15 degrees) reaches 52.5%, which is larger than the 42.4% of small-angle boundaries (lower than 15 degrees); thus, the plasticity can be improved.

### 3.2. Hardness of MLS

To characterize the microstructure of MLS, the hardness was also measured. The hardness of the ML layer can reach 385.2 HV, which is higher than 310 HV in the ML_1_ layer. It is attributed to the large block Cr_23_C_6_ distributed in the ML layer. The hardness of the large block Cr_23_C_6_ can reach 1400 HV.

### 3.3. Results of Tensile Tests

**Figure 4:**
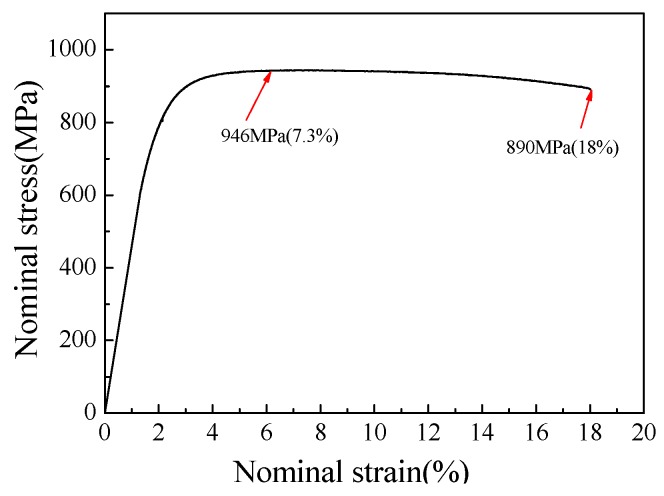
Engineering stress–strain curve of a multilayered steel (MLS) tensile specimen.

**Figure 5:**
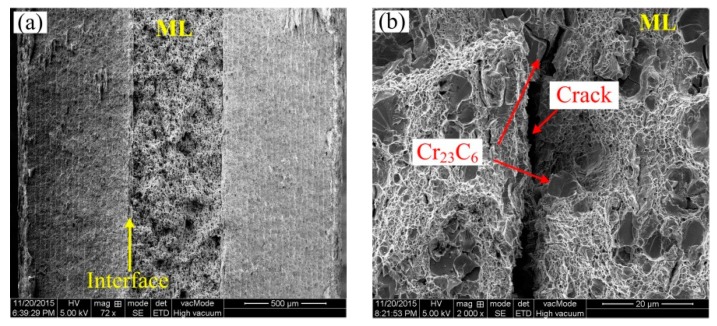

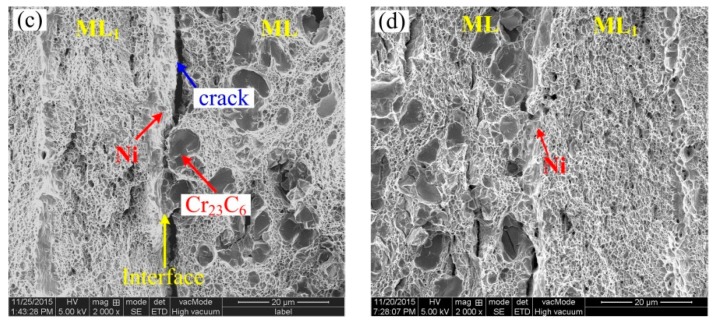
Fracture surface of MLS tensile specimen. (**a**) the macro-fracture surface, (**b**) the magnification of ML in Figure 5a, (**c**) the magnification of the interfaces of the ML and Ni layer and the ML_1_ and Ni layer in Figure 5a, (**d**) the magnification of the interfaces between the ML_1_ layer and the Ni layer in two sides of MLS.

Figure 4 presents the engineering stress–engineering strain curves of a MLS tensile specimen. The ultimate tensile strength and the maximum tensile elongation reaches 946 MPa and 18%, respectively. To reveal the tensile fracture behavior, the tensile fracture surface is shown in Figure 5. Figure 5a shows the macro-fracture surface; from Figure 5a, we can see that the normal fracture is induced at the middle location of the tensile specimen. However, some shear feature along the 45-degree direction can be seen at the edge of the specimen. The fracture surface of the middle ML layer in Figure 5a is magnified in Figure 5b, in which the cracking of the large block carbides and dimple in the matrix dominate the fracture surface of the middle ML layer. Figure 5c presents the magnification of the interfaces of the ML and Ni layer and the ML_1_ and Ni layer. It can be found that delamination can be found in the interface between the middle large ML layer and the Ni layer. It is related to the different hardness. This is because there are lots of large hard block Cr_23_C_6_ phases distributed in the ML layer near the Ni layer, and the thickness of the middle ML layer reaches 575 μm. The middle higher hardness ML layer with a larger thickness of 575 μm makes large strain incompatibility in the interface between the ML layer and the Ni layer. However, in the interface between the ML_1_ layer and the Ni layer in two sides of MLS, amounts of deformation and diffusion can be produced at the interface, and local brittle carbide cracking and plastic dimples are all produced in the interface in Figure 5d. It means that better deformation can be obtained at the interface between the Ni layer and the ML_1_ layer. From Figure 5c, local delamination is produced in the interface between the ML layer and the Ni layer, which leads to lower tensile ductility in Figure 4. According to Ref. [7], the combined quality of the interface has a direct effect on the ductility of the MLS.

### 3.4. Results of Bending Tests

**Figure 6:**
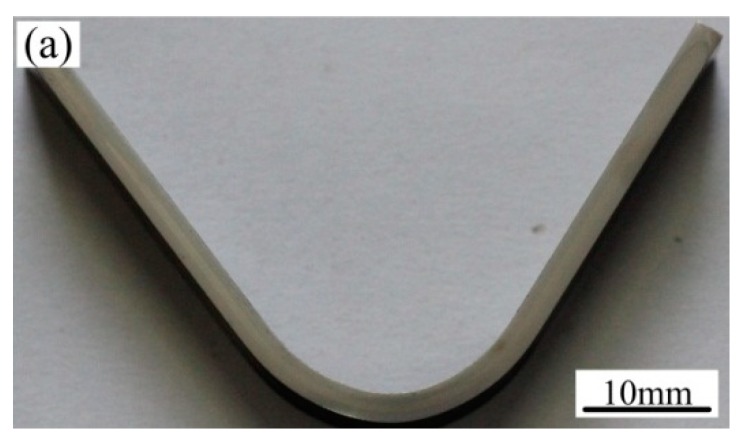

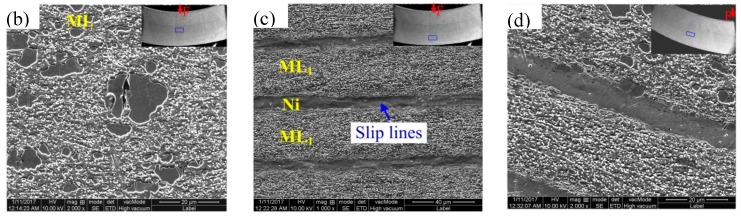
Macro-feature and SEM morphology of the bending specimen after bending to 136 degrees. (**a**) Macroscopic bending morphology, (**b**–**d**) SEM bending features.

When the MLS bending specimen was bent to 136 degrees, a macro-bending feature is shown in Figure 6a. The average maximum bending load F_bb_ is 3.8 KN, and the bending strength can be calculated by the following Formula (1). In Formula (1), σ_bb_ is the bending strength, F_bb_ is the maximum bending load, Ls is the loading span, w is the factor of specimen’s cross-section, and w = bh^2^/6 (b is the width of the bending specimen, h is the thickness of the bending specimen). By using an F_bb_ of 3.8 KN, loading span (Ls) of 28 mm, width of the bending specimen (b) of 10 mm, and thickness of the bending specimen (h) of 2.25 mm, the average bending strength σ_bb_ can be calculated as 3153 MPa.

(1) $$\sigma_{\mathrm{bb}}=\frac{F_{\mathrm{bb}}L_{s}}{4W}$$

The detailed bending processes will be analyzed by micro-observed features in Figure 6b–d.

Figure 6b–d present the magnification of various locations when the MLS specimen was bent to 136 degrees, the location corresponding to the bending specimen is arranged in the sub-figure of the upper right corner of the image map. Figure 6b,c show the magnification of the middle layer and the side layer in maximum bending moment of the specimen, while Figure 6d shows the magnification of the side layer far from the maximum bending moment of the specimen. From Figure 6b, only a local crack can be found in the largest hard block Cr_23_C_6_ phase. However, in the Figure 6c, no cracks can be found, and more slip lines appear in the Ni layer. In Figure 6d, no cracks can be produced in the Ni layer and ML_1_ far from the maximum bending moment of the specimen. 

**Figure 7:**
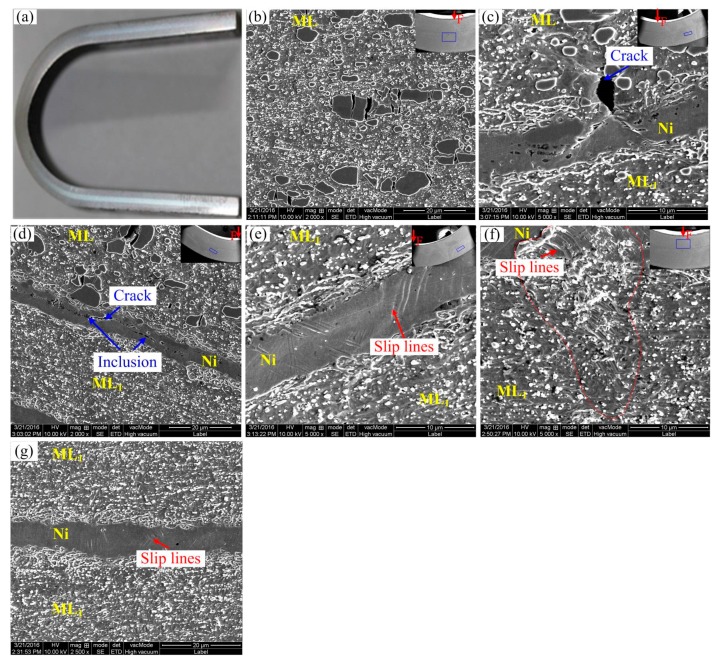
Macro-feature and SEM morphology of the bending specimen after bending to 180 degrees. (**a**) Macroscopic bending morphology, (**b**–**g**) SEM bending features.

To further reveal deformation behavior after bending to 136 degrees, the MLS specimen was also bent to 180 degrees, as shown in Figure 7. There are no macro-cracks in the outer side of the bending parts. To carefully observe micro-cracking features in the maximum bending moment and near, the part is observed in SEM, as shown in Figure 7. In Figure 7, the location corresponded to the bending specimen is arranged in the sub-figure of the upper right corner of the image map, and marked by the blue area of the upper right corner figure. From Figure 7b, in the largest bending moment part of the middle ML layer (on the blue area of the upper right corner figure), a lot of block carbide was directly cracked or cracked along the boundary between the block carbide and the matrix because of incompatible deformation. Figure 7c shows the cracking hard block carbide phase in the middle ML layer near the Ni layer; after further loading, the stress concentration was formed at the crack tip. When the stress reaches a certain degree, the crack is extended to the Ni layer. More plastic deformation was produced at the Ni layer and the matrix ahead of the crack. An obvious slip line in the Ni layer was distributed along the 45-degree direction in front of the crack. From this phenomena, the presence of the Ni layer delayed crack propagation. In Figure 7d, local cracks were produced at the Ni layer. It may be concerned with the element diffusion from the middle ML layer, which results in some fine carbide existing in the Ni layer. However, in Figure 7e–g, even though the specimen was bent to 180 degrees, in the ML_1_ and the Ni layer on both sides of MLS, no crack can be found, only plastic deformation traces, while some slip lines occur in different degrees. It shows that the flexural capacity of the specimen is still relatively satisfied, although in the middle harder ML layer, amounts of brittle block carbides were cracked. It should be attributed to the large brittle carbide particles due to high carbon.

### 3.5. Results of In-Situ Tensile Tests

**Figure 8:**
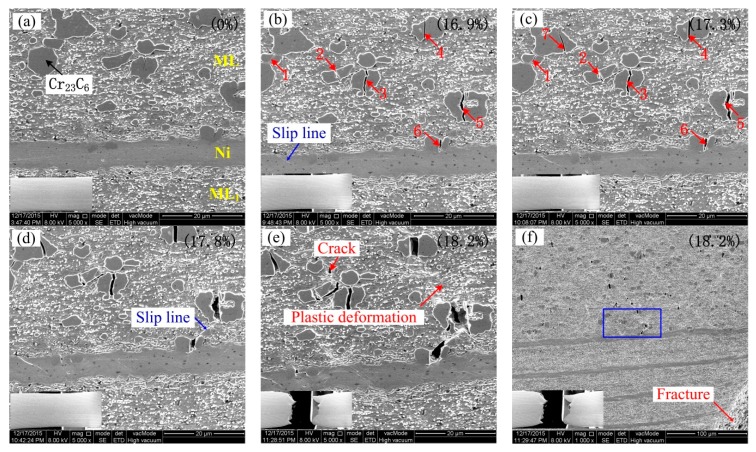
SEM features and cracking of carbides observed at various tensile elongations, (**a**) 0, (**b**) 16.9%, (**c**) 17.3%, (**d**) 17.8%, (**e**,**f**) 18.2%.

To observe the crack initiation and propagation processes during the tensile loading, in-situ SEM tensile tests were done. Figure 8 reveals the cracking of the carbides during loading processes with the insets in each part showing macro-features. Figure 8a shows the feature before loading. When the tensile elongation of the specimen reaches 16.9%, a necking is just produced at the tensile gauge, and a short and small crack is produced in the block carbide particles of the ML, as indicated by the red arrows 1, 2, 3, 4, 5, and 6 in Figure 8b. Moreover, a short slip line is produced in the Ni layer, which is marked by a blue arrow. When the tensile elongation is increased to 17.3%, another crack 7 is cracked in the carbide particle except for the further growth of the cracks 1, 2, 3, 4, 5, and 6, as shown in Figure 8c. With a further increase in elongation of 17.8%, as shown in Figure 8d, seven cracks further grow and become wider, but they are interrupted by the boundary of the carbides. However, an obvious shear slip line is produced between crack 5 and crack 6. Finally, as the tensile elongation reaches 18.2% (Figure 8e,f), even at the moment of fracture, only very small cracks can be induced in the ML layer, and local plastic deformation is still produced in the ML (Figure 8e,f). Finally, the specimen fractures in the necking zone, as shown in Figure 8e,f. After the specimen has fractured, some cracked carbides can propagate and further connect each other.

**Figure 9:**
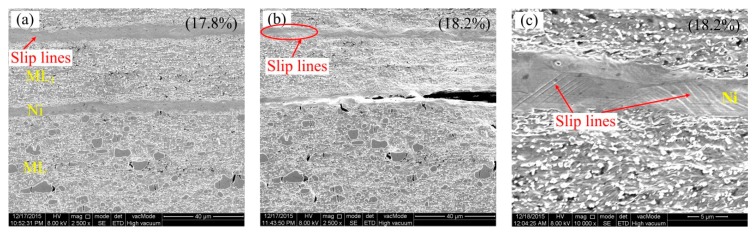
Deformation of the Ni layer observed at various tensile elongations, (**a**) 17.8%, (**b**,**c**) 18.2%.

Moreover, besides the cracking processes of the carbides being observed, the slip deformation of the Ni layer near the fracture is also observed in Figure 9. From Figure 9a, in the Ni layer, a large number of slip bands are produced, but the brightness of the slip band is still shallower. When the tensile elongation reaches 18.2%, at the moment of fracture, as shown in Figure 9b, the slip bands increase and become deeper. The red area in Figure 9b was enlarged in Figure 9c; the direction of the slip bands in the Ni layer is along 45 degrees, which is 135 degrees to the normal tensile stress direction. It is because the Ni in the Ni layer belongs to the Face Center Cubic (FCC) metal, and there are multiple slip systems with good plastic deformation. The deformation of the thinner Ni layer is also limited due to the restriction of the brittle layer near the Ni layer.

**Figure 10:**
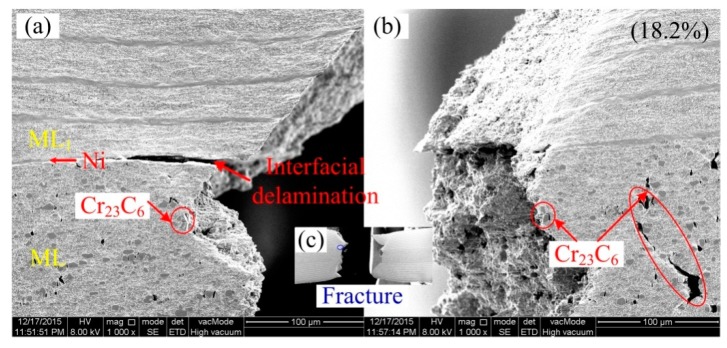
Fracture surfaces of multilayered steel (MLS) in-situ tensile specimen (**a**) one fracture surface, (**b**) matched fracture surface.

At the moment of fracture, the delamination is produced at the interface between the Ni layer and middle ML. The delamination phenomena can be obviously presented in the fracture surface of an in-situ tensile specimen, as shown in Figure 10. Two matched fracture surfaces can reveal the carbide cracking and relationship between in-situ observed cracking processes and fracture surface. Amounts of carbides cracking in the middle ML induce the lower tensile elongation. However, dimple fracture dominates the fracture in the Ni layer and ML_1_ layer. 

## 4. Discussion

### 4.1. The Effect of Block Brittle Carbides on Fracture Processes

From Figure 5, Figure 7 and Figure 8, for the middle high carbon ML layer with a thickness of 575 um, whether in tensile, in-situ tensile, or bending processes, the same phenomena can be seen. When the specimen was loaded to the certain applied load or certain elongation, the cracks were suddenly initiated in block brittle carbides in ML; finally, the cracks connect and the specimens fracture. So, the fracture is micro-crack damage-induced fracture mode, which is consistent with the fracture mode of TiAl alloys in Ref. [14]. During tensile or bending processes, only the cracks were produced at the larger block carbides in ML; no cracks can be produced in ML_1_ or finer carbides in ML. It may be because the brittleness of larger block carbides is larger than that of finer carbides in ML and ML_1_. The hardness of the larger block carbide can reaches 1400 HV, which directly contributes to the larger brittle of ML. So, if the larger block carbides can be controlled to finer size, the tensile ductility and the bending deflection will be improved.

### 4.2. The Effect of Ni Layer on Tensile Elongation and Bending Flection

According to Ref. [15,16], the Ni layer can reduce the diffusion of Cr elements to the interface, and Cr has the strong ability of absorbing carbon. Referring to [6,11,17], although the Ni layer cannot completely inhibit C diffusion, the Ni layer can significantly reduce the diffusion distance of the C element due to the low diffusion coefficient C in Ni. It avoids the direct contact between the high carbon martensite layer and low carbon martensite layer. Since large C concentration difference leads to the migration of C elements, thus, amounts of Cr carbides will be produced in the interface, which decrease the plastic of the interface [13]. In this paper, in the interface between the ML and Ni layer, and the interface between the ML_1_ and Ni layer, various fracture modes and cracking behaviors were shown in Figure 7, Figure 8, Figure 9 and Figure 10. In the interface between the ML1 and Ni layer, no cracks can be found, while amounts of slip bands contribute final dimple fracture modes. During interpreting the superior strength-ductility mechanism of laminated or gradient composites, several deformation mechanisms were proposed, including the delayed necking [18], orderly plastic deformation [19], enhanced strain-hardening capacity [20,21], ideal confinement by laminated or gradient microstructure [22], etc. The similarity of these mechanisms is that they can be linked by “strain non-localization”; in other words, the heterogeneity of the microstructure effectively suppresses the degree of strain localization. Based on the above presented mechanism, in this paper, higher strength ductility is also related to “strain non-localization”. Under the tensile loading, the local brittle carbide phase cracking in the middle martensite layer firstly occurred, and it can continue to carry the loads transmitted by the adjacent ductile Ni layer. The observed strength-ductility mechanism is a result of constrained crack propagation behavior and strain non-localization imparted by the multilayered structure.

However, in the interface between the ML and Ni layer, delamination phenomena and some local larger brittle carbides can be found. It means that the diffusion degrees of C in two different layers to the Ni layer are different due to different carbon contents in the ML and ML_1_ layers. Currently, it can also be related to the larger thickness of the middle ML. It can be identified in [23]. In [23], the reasonable choice of the material and the thickness for the middle layer will effectively improve the combined strength of the composite plates.

## 5. Conclusions

In this paper, the microstructure and mechanical properties of multilayered steel (MLS) are evaluated by SEM, EBSD, tensile tests, a bending test, and an in-situ tensile test. The obtained results are summarized as follows.

(1) A special MLS can be manufactured using three different materials by the accumulative roll-bonding method. It is composed of the middle and thicker high-carbon martensite layer and the multilayer composite layers at two sides. The middle and thicker martensite layer is composed of high-carbon martensite steel, of which the microstructure is composed of ferrite base metal and Cr_23_C_6_ and Cr_7_C_3_ carbides distributed in the matrix. The multilayer composite layer in the two sides of MLS is alternately composed of 19 middle carbon martensile layers (ML_1_) and 19 pure Ni thin layers distributed in two sides of the middle ML. ML_1_ is composed of a ferrite base metal and Cr_23_C_6_ and Cr_7_C_3_ carbides distributed in the matrix. The microstructure of the Ni layer is single austenite; besides, some diffusion of element Ni, Fe, Cr in the bonding interface can be found.

(2) The MLS can reach the ultimate tensile strength of 946 MPa and the maximum tensile elongation of 18%. Bending tests were conducted to investigate the formability of MLS. When the MLS was bent to 136 degrees, the bending strength can reach 3153 MPa, and the deflection can reach 22 mm. Even though the specimen was bent to 180 degrees, no macro-crack still can be found, only plastic deformation traces, and some slip lines occur in different degrees.

(3) During tensile processes, the cracks were firstly initiated in block carbide particles (Cr_23_C_6_) of the ML. With the further increase of applied load, the Cr_23_C_6_ block phase is snapped and the number of cracks increases; finally, the cracks connect and the specimens fracture. 

(4) In the whole fracture process, both the Ni layer and the metal on both sides (MLS) have good plasticity, and the plastic deformation plays a crucial role in the plasticity of the MLS. The middle and larger martensite layer (ML) becomes the weakest link of tensile fracture and causes the interfacial delamination of the MLS. The cracking of the large block carbides and the dimple in the matrix dominates the fracture surface of the middle ML layer. However, the dimple fracture dominates the fracture in the Ni layer and ML_1_ layer, and the interface combination between the Ni layer and ML_1_ layer is good.

## Figures and Tables

**Table 1:** Main chemical composition of multilayered steel (mass %).

Materials	Compositions (mass %)						
	C	S	P	Si	Mn	Cr	Mo
High carbon martensite layer	1.07	0.007	0.024	0.32	0.31	16.63	0.074
Middle carbon martensite layer	0.32	0.007	0.028	0.37	0.55	11.73	~

**Table 2:** Main chemical composition of carbides in Figure 3d.

Location	C	Cr	Fe	Mo	Mn	Si
1	40.2	33.2	24.7	1.3	0.5	0.1
2	26.0	12.8	59.6	0.4	0.5	0.7
3	37.9	36.3	25.0	~	0.5	0.7
